# Inconsistent Wassermann Findings in Mother and Infant

**Published:** 1914-08

**Authors:** 


**Inconsistent Wassermann Findings in Mother and Infant.** Cassoute, Gaz. des Prat., May 1, 1914, cites a case in which two tests showed positive in the infant and negative in the mother. The latter denied syphilis and was apparently non-syphilitic. The infant did not show the ordinary signs but was atrophic and had enlarged liver and spleen and improved under mercury, the mother continuing to nurse it without sign of being herself infected. He quotes Knopfelmacher and Lehndorffer as having examined 32 serums of mothers apparently healthy but who had borne syphilitic children. 18 of the maternal serums reacted positively.

